# Quantifying emergent drug-resistant TB using laboratory data and record-linkage methods

**DOI:** 10.5588/ijtldopen.26.0043

**Published:** 2026-07-13

**Authors:** C.H. Rhea, F. Maruri, Y. Ghebrekristos, G. Amorim, H. Cox, K. Dheda, T.R. Sterling, R. Warren, Y.F. van der Heijden

**Affiliations:** 1 Division of Epidemiology, Vanderbilt University School of Medicine, Nashville, TN, USA;; 2 Vanderbilt Tuberculosis Center, Nashville, TN, USA;; 3 Division of Infectious Diseases, Department of Medicine, Vanderbilt University School of Medicine, Nashville, TN, USA;; 4 DSI-NRF Centre of Excellence for Biomedical Tuberculosis Research, and SAMRC Centre for Tuberculosis Research, Division of Molecular Biology and Human Genetics, Faculty of Medicine and Health Sciences, Stellenbosch University, Tygerberg, South Africa;; 5 National Health Laboratory Service, Greenpoint Tuberculosis Laboratory, Cape Town, South Africa;; 6 Department of Biostatistics, Vanderbilt University Medical Center, Nashville, TN, USA;; 7 Division of Medical Microbiology, Department of Pathology, University of Cape Town, Cape Town, South Africa;; 8 Institute of Infectious Disease and Molecular Medicine, University of Cape Town, Cape Town, South Africa;; 9 The Burnet Institute, Melbourne, VIC, Australia;; 10 Centre for Lung Infection and Immunity, Division of Pulmonology, Department of Medicine and UCT LUNG Institute & South African MRC/UCT Centre for the Study of Antimicrobial Resistance, University of Cape Town, Cape Town, South Africa;; 11 Faculty of Infectious and Tropical Diseases, Department of Immunology and Infection, London School of Hygiene and Tropical Medicine, London, UK;; 12 The Aurum Institute, Johannesburg, South Africa.

**Keywords:** tuberculosis, rifampicin-resistant TB, fluoroquinolone resistance, linking patient results

## Abstract

**BACKGROUND:**

Clinicians, public health workers, and researchers must link multiple laboratory results to individual patient records to identify emerging drug-resistant TB.

**OBJECTIVE:**

Determine the proportion of individuals with rifampicin-resistant TB (RR-TB) who developed fluoroquinolone resistance using different data matching methods.

**DESIGN:**

Data from the National Health Laboratory Service (NHLS) from the Western Cape Province, South Africa (January 2008 to June 2015), were used to identify patients with RR-TB who developed fluoroquinolone resistance after initial susceptibility. We used four methods to link patient results: A) exact match on NHLS patient number; B) exact match on surname, first name, and date of birth; C) custom algorithm (Stellenbosch University) using approximate matches for patient-identifying variables; and D) custom iterative approximate linkage developed for this study. We manually reviewed matches identified by each method.

**RESULTS:**

We identified 30,381 laboratory results showing rifampicin and fluoroquinolone susceptibility. Estimates of resistance development were 4% using methods A and B, and 8% using methods C and D. Across methods, 688 unique patients who developed fluoroquinolone resistance were identified, 598 of which were correct by manual review. Method D identified 587 (98%) of these correctly matched patients, the largest proportion across these methods.

**CONCLUSION:**

Customised approximate linkage approaches maximised correct patient matches.

Despite ongoing efforts to shorten therapy for both drug-susceptible TB (DS-TB) and drug-resistant TB (DR-TB), recommended minimum treatment durations (4 months for DS-TB and 6 months for DR-TB) necessitate substantial longitudinal follow-up.^1–3^ TB patients require routine clinical and laboratory monitoring throughout treatment, and may experience recurrent episodes of TB, particularly in high-burden settings.^4^ Public health surveillance and epidemiologic research often rely on programmatic data from centralised laboratory databases or patient data from regional or national registries rather than curated cohorts.^5–7^ Among concerns related to programmatic data, such as security, quality, and bias, the reliability or absence of unique patient identifiers poses a significant challenge for linking records during longitudinal care of TB patients.^7–10^ Reliable data linkage is essential for follow-up and treatment monitoring, as patients undergo multiple instances of laboratory testing, including assessment of *Mycobacterium tuberculosis* cultures and monitoring for drug toxicities. This generates longitudinal clinical and laboratory data points for each patient, which clinicians use to inform treatment decisions and assess progress over time.

Drug resistance threatens TB control, particularly in settings with a high burden of HIV.^11^ Monitoring a TB patient’s drug-susceptibility profile is essential for initiating or modifying their treatment plan. Drug resistance may be acquired during treatment, often arising from inappropriate or incomplete treatment (e.g., loss to follow-up, interrupted drug supply, and patient migration).^12^ In endemic settings, patients may also be reinfected with *M*. *tuberculosis* strains that carry additional drug resistance.^13^ Detecting emerging drug resistance during treatment is critical to ensuring timely modifications to therapy. Achieving this requires accurate linkage of follow-up drug-susceptibility results to a patient’s record. However, linking patient health information remains an ongoing challenge in many high-burden settings, particularly where systems lack reliable unique patient identifiers. While strategies have been developed to address these challenges, no studies have compared how different approaches affect the linkage of longitudinal data needed to detect emerging drug resistance.

For this study, we evaluated four linkage strategies and determined how efficiently they match individual laboratory results belonging to the same patient and identify emerging fluoroquinolone resistance – an important class of medications used in TB treatment.^14,15^ We compared the accuracy of each method and examined where failures or discrepancies occurred. These findings underscore the importance of assessing errors, while highlighting features of effective linkage strategies.

## METHODS

**Figure 1.**
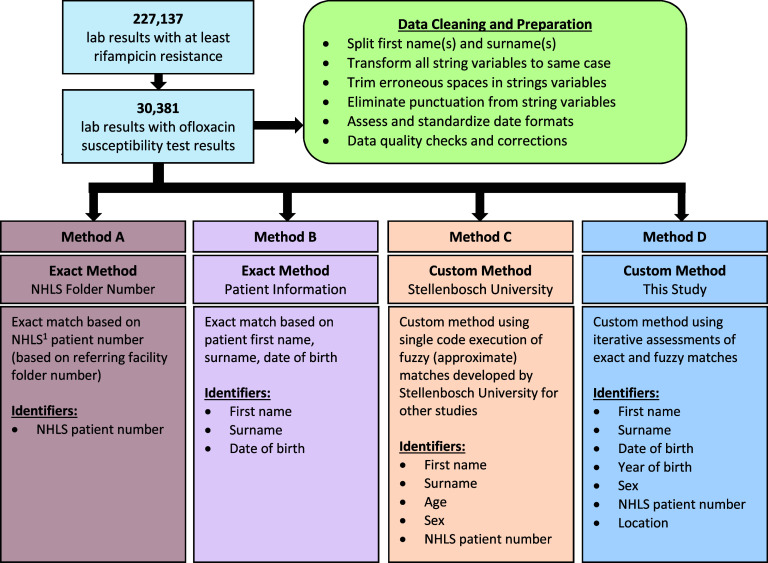
Study diagram showing selection of laboratory results, data preparation steps, and details of the four patient-matching strategies. NHLS = National Health Laboratory Service.

National Health Laboratory Service (NHLS) data from the Western Cape Province, South Africa (January 2008 to June 2015), were used. The dataset contained individual laboratory reports with drug-susceptibility results, and analyses were restricted to reports that included fluoroquinolone susceptibility information. Routine phenotypic testing for ofloxacin sensitivity was used to characterise fluoroquinolone susceptibility. We used four methods to link results: A) exact match on NHLS patient number based on referring facility folder number; B) exact match on surname, first name, and date of birth; C) custom approach using approximate (fuzzy) matches for patient surname, first name, age, sex, and NHLS patient; and D) custom approach using iterative assessments of approximate matches on patient surname, first name, date of birth, year of birth, sex, NHLS patient number, and location (Figure 1).

Before executing linkage methods, we confirmed retained NHLS results included both rifampicin and ofloxacin susceptibility, then performed cleaning steps. This included standardising string variables by case formatting, eliminating punctuation and erroneous spaces, splitting first and surnames, harmonising date formats, and checking for internal consistency. For methods A and B, string and date variables had to match exactly. Method C used a custom programme developed at Stellenbosch University that incorporated exact and approximate linkage involving first name, surname, age, sex, and NHLS patient number. Method D was developed for this study and involved starting with exact matches of first name, surname, and date of birth. After manual review of matches within the list, multiple rounds of new linkage commands incorporating both exact and approximate combinations of identifying variables were executed, each round followed by manual review for correct matches, failed linkages, and incorrect matches (see Supplementary Data for code).

For each method, we executed the following procedures: first, determined the total number of unique patients that could be identified within the dataset; second, among the uniquely identified patients, determined the number with rifampicin-resistant TB (RR-TB); and third, among RR-TB patients, determined the number with emerging ofloxacin resistance – defined as at least one confirmed phenotypic ofloxacin-sensitive drug-susceptibility result, followed by an ofloxacin-resistant result. We then manually reviewed matches for RR-TB patients who developed ofloxacin resistance to confirm they belonged to a single patient. One investigator reviewed available information, including first name, surname, date of birth, sex, clinic, folder, and identification numbers to determine correct linkage, particularly in the presence of discrepancies (e.g., name spelling variations, transposed first and surname, or date of birth entry errors). We then determined the number and percentage of correct (i.e., identifiable information matches between records; correctly linked multiple results from a single patient), uncertain (i.e., some discrepancies or missing information prevented confident assignment of correct or incorrect match), and incorrect matches (i.e., identifiable information did not match between records; incorrectly linked results from multiple patients into a single record). Correct matches served as the reference for evaluating the performance of each linkage method, including identifying instances in which methods failed to link all records belonging to a patient. Independent reviews were conducted by another researcher repeating the linkage steps and manual review to verify outcomes from the original analysis. All statistical analyses were performed in STATA-BE version 17.0 (STATA Corporation, College Station, TX, USA).

### Ethical statement

Ethics approvals and waiver of informed consent were granted by Vanderbilt University Institutional Review Board (IRB Number 131289), Human Research Ethics Committees at University of Cape Town (Reference Number 614/2014), and Stellenbosch University (Reference Number N14/08/106).

## RESULTS

During the study period, NHLS data from the Western Cape Province included 227,317 laboratory records with rifampicin susceptibility results. Use of linkage strategies was limited to the 30,381 records that also contained ofloxacin-susceptibility results (Figure 1).

Table 1 presents overall results, including the number of unique patients and number of RR-TB patients, and longitudinal results, including the number and percentage of RR-TB patients who developed ofloxacin resistance identified by each method. Among 30,381 laboratory results, method A identified 20,492 unique patients and 10,225 RR-TB patients, of whom 449 (4.4%) developed ofloxacin resistance, representing 12.9% of the 3,484 patients who had at least two ofloxacin susceptibility results. Method B identified 21,079 unique patients and 10,188 RR-TB patients, of whom 453 (4.4%) developed ofloxacin resistance, representing 14.5% of 3,132 with at least two ofloxacin-susceptibility results. Method C identified 17,028 unique patients and 8,178 RR-TB patients, of whom 627 (7.7%) developed ofloxacin resistance, which is 14.2% of 4,342 with at least two ofloxacin-susceptibility results. Finally, method D identified 16,802 unique patients and 7,949 RR-TB patients, of whom 603 (7.6%) developed ofloxacin resistance, which is 14.7% of 4,080 with at least two ofloxacin-susceptibility results.

**Table 1.** Comparison of unique patient identification approaches and matching methods to determine ofloxacin-resistant TB development among patients with rifampicin-resistant TB (RR-TB).

	Method A, n (%)	Method B, n (%)	Method C, n (%)	Method D, n (%)
Overall results				
Uniquely identified patients (n)	20,492	21,079	17,028	16,802
Uniquely identified patients with RR-TB (n)	10,225	10,188	8,178	7,949
Longitudinal results				
Patients with RR-TB and ofloxacin sensitivity, then resistance, out of all with RR-TB (n, %)**^A^**	449 (4.4)	453 (4.4)	627 (7.7)	603 (7.6)
Patients with RR-TB and ≥2 ofloxacin-susceptibility results (n)	3,484	3,132	4,342	4,080
Patients with RR-TB and ofloxacin sensitivity, then resistance, out of those with ≥2 ofloxacin-susceptibility results (n, %)**^B^**	449 (12.9)	453 (14.5)	627 (14.2)	603 (14.7)

Matching methods, A: exact by National Health Laboratory Service patient number; B: exact by patient surname, first name, and date of birth; C: custom developed by Stellenbosch University; D: custom developed by this study.

A Percentage based on the total number of uniquely identified patients with RR-TB.

B Percentage based on the total number of uniquely identified patients with RR-TB who had at least two ofloxacin-susceptibility results.

**Figure 2.**
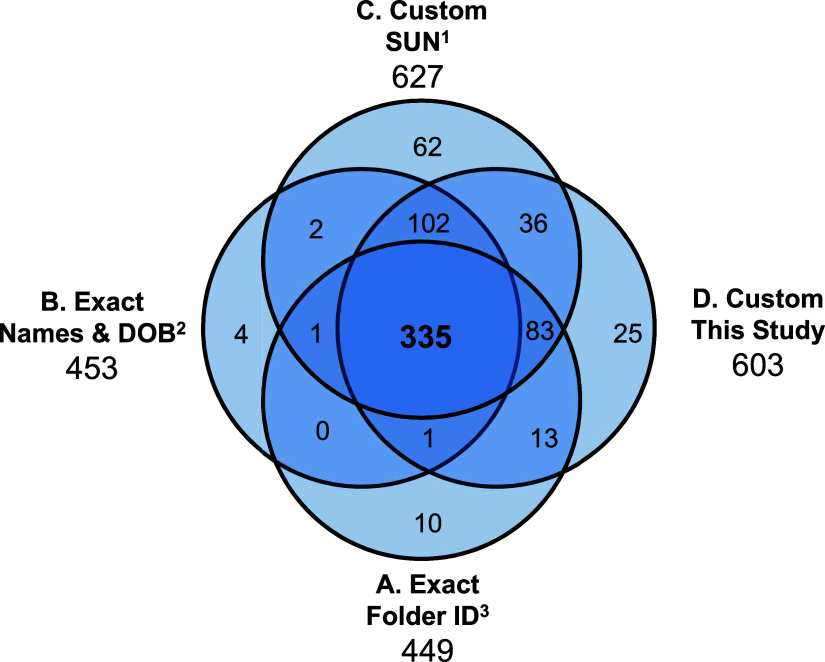
Venn diagram showing the relationship of four matching approaches used to determine the number of patients with multidrug-resistant TB who developed ofloxacin resistance (total of 688 patients identified by any approach). Not shown: A + C = 6; B + D = 8. SUN = Stellenbosch University; DOB = date of birth; ID = identification (National Health Laboratory Service patient number).

We found 688 total possible RR-TB patients who developed ofloxacin resistance when we combined all patients identified by any of the linkage methods (Figure 2). Table 2 displays results from manual review that identified 598 correct, 37 uncertain, and 53 incorrect linkages. Exact methods each identified more than half of total matches (A = 65%, B = 66%) and three-quarters of correct matches (A = 72%, B = 74%), with method A also linking one-quarter (28%) of incorrect matches. This resulted in lower sensitivity (A = 72%, B = 74%), but high positive predictive value (PPV) (A = 96%, B = 98%). Custom approaches identified a larger proportional of total matches (C = 91%, D = 87%) and most correct matches (C = 94%, D = 98%). Method C also made many of the uncertain (76%) and incorrect (70%) matches, while method D linked fewer (38% and 4%, respectively). This resulted in higher sensitivity (C = 94%, D = 98%) and PPV (C = 90%, D = 98%).

**Table 2.** Outcomes of manual review of any discrepancies among 688 patients identified by any one of the matching methods with an ofloxacin-sensitive isolate followed by an ofloxacin-resistant isolate.

	Method A, n (%)	Method B, n (%)	Method C, n (%)	Method D, n (%)	Total n
Results of manual linkage review**^A^**					
Correct	431 (72)	442 (74)	562 (94)	587 (98)	598
Uncertain	3 (8)	5 (14)	28 (76)	14 (38)	37
Incorrect	15 (28)	6 (11)	37 (70)	2 (4)	53
Total	449 (65)	453 (66)	627 (91)	603 (87)	688
Sensitivity and positive predictive value (PPV) per method**^B^**					
Sensitivity	72%	74%	94%	98%	
PPV	96%	98%	90%	98%	

Matching methods, A: exact by National Health Laboratory Service patient number; B: exact by patient surname, first name, and date of birth; C: custom developed by Stellenbosch University; D: custom developed by this study.

Sensitivity_Method_ = TP/(TP + FN). i) True positive (TP) = number of correct matches identified within each method. ii) False negative (FN) = number of true patient matches (n = 598) that were not identified within each method.

PPV_Method_ = TP/(TP + FP). iii) True positive (TP) = number of correct matches identified within each method. iv) False positive (FP) = combined number of uncertain and incorrect matches identified within each method.

A Manual record validation determined whether matching strategy correctly or incorrectly identified unique patients who developed ofloxacin resistance, or if sufficient data were unavailable (uncertain). Percentages shown are per row.

B The 598 total patients identified as correct matches by any one of the linkage methods are considered the total number of true patients to be identified for sensitivity calculations.

**Figure 3.**
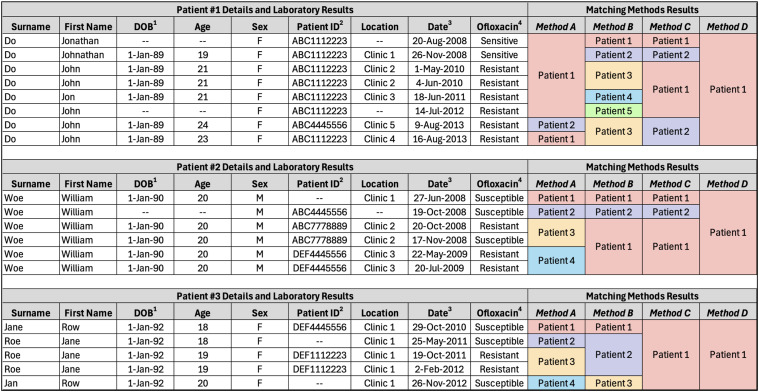
Illustration of the four matching methods attempting to link patient records together. This figure includes three sets of de-identified laboratory results that each belong to a single, unique patient. Using the matching methods under evaluation in this study, we can determine the number of unique patients that were identified among these results. De-identified laboratory results obtained by anonymising surname, first name, date of birth, and patient ID; dates were also shifted as described in the All of Us study to ensure de-identification while retaining order.^16^ Matching methods, A: exact by National Health Laboratory Service (NHLS) patient number; B: exact by patient surname, first name, and date of birth (DOB); C: custom developed by Stellenbosch University; D: custom developed by this study. Patient ID = NHLS patient number; date = specimen collection date; ofloxacin = ofloxacin drug-susceptibility result.

Figure 3 illustrates the four linkage methods using three de-identified patient examples. Each set of laboratory results belongs to a single patient, but due to data entry errors and missing information, each method linked a different combination of records. This led to inconsistent identification of total unique patients and hindered the ability to detect changes in drug resistance. Independent review replicating the matching analysis and manual review confirmed these results.

## DISCUSSION

Our study found custom linkage methods outperformed exact approaches by more accurately combining longitudinal records, identifying more RR-TB patients who developed ofloxacin resistance. Exact methods (A and B) linked records together in a way that resulted in a larger number of total unique and RR-TB patients due to failed linkages, thereby capturing fewer who developed ofloxacin resistance compared to custom methods (C and D). Manual review of the 688 identified RR-TB patients who developed ofloxacin resistance by at least one method revealed exact methods had lower sensitivity compared to custom methods. Despite their high PPV, exact methods often failed to link all results belonging to the same patient due to variations in the identifiers used to link records. As a result, they were less effective at detecting longitudinal changes such as emergent ofloxacin resistance. By contrast, custom methods accommodated for inconsistent and incomplete data, allowing for more accurate, comprehensive linkage, increasing sensitivity while maintaining high PPV, reducing the risk of missing important clinical developments.

The patient examples in Figure 3 illustrate how the linkage methods can differ both in the number of unique patients identified at a single point in time and the detection of drug resistance acquisition. Patient #1 had inconsistencies in first name spelling, missing dates of birth, and was assigned multiple patient IDs. As a result, the exact methods treated multiple of these results as belonging to separate individuals. Method B, for instance, identified five unique patients and failed to capture ofloxacin resistance development. Next, Patient #2 had consistent name spelling and more reliable date of birth entry, but still had multiple patient IDs assigned, leading method A to identify four unique patients where again, the development of ofloxacin resistance was missed. Finally, Patient #3 highlights a common data entry error observed – transposition of patient surname and first name. This, combined with missing or duplicate patient IDs, prevented the exact methods from linking records. Conversely, custom methods were better equipped to handle these limitations and resulted in more complete and accurate linkages. Notably, method D successfully linked all records across each of the three patients and captured the development of ofloxacin resistance. Accurate linkage of patient records is essential, not only for data quality, but patient care. Missing critical clinical developments, such as acquired drug resistance due to poor linkage, can delay treatment modifications and adversely impact patient outcomes.^17^

Figure 3 further explains why exact methods identified more unique patients and RR-TB patients, but fewer who developed ofloxacin resistance, compared to custom methods. Due to data inconsistences and rigid matching criteria, the exact methods often failed to link all records belonging to a single individual and instead generated multiple unique patient records for the same person. For example, Patient #3 in Figure 3 – method A identified four unique individuals and method B identified three, despite these records belonging to one person. This artificially inflated the total number of unique patients identified by the exact methods and missed longitudinal changes. In contrast, custom methods C and D successfully linked these results into a single patient record, accurately capturing resistance development. Results in Table 2 also showed that false linkages can be made, which could result in negative downstream effects if such linkages are not reviewed before clinical decisions are made.

High-TB-burden settings such as South Africa have a large volume of laboratory results that need to be linked to individual patient records, where manual tracking and verification is often not possible.^18^ One main reason data linkage and reliable follow-up for patient evaluation differed across methods is the variability in patient data. This can result from discrepant information provided by patients (i.e., patients may provide different names or date of birth) or variations in the way information is recorded (e.g., name spelling variations, transposition of first and surname, and date of birth transposition of day and month). Data entry errors and missing information have also been noted by other studies evaluating data quality and linkage methods.^5,8,19,20^ Lacking reliable, accurate patient identification information makes data linkage more challenging, even with custom linkage strategies. These findings highlight the need to train and assess health care staff on the importance of accurate data collection and management for the purposes of data linkage and provide them with resources to conduct regular data quality evaluations.^18^ Further, enhancements such as standardisation of data fields, use of technology, and implementation of unique identifiers (e.g., biometric, assignment of identification despite foreign nationality) could further minimise entry and linkage errors, including both failures to link patient data and making false linkages.^5,21^ These efforts will support accurate data entry and enhance quality, ultimately improving the success of data linkage strategies such as those assessed in our study.

Our findings highlight longstanding challenges in matching longitudinal TB patient data and the constraints of exact methods, particularly in settings without universal patient identification – a gap that exists today.^22^ Data analysed in this study were collected between 2008 and 2015, and while analyses and dissemination were delayed, this well-characterised dataset enabled a robust evaluation of laboratory linkages and supports continued clinical and epidemiologic relevance of the findings. Our study also had limitations, namely the use of historic data, as the rates of fluoroquinolone use in TB treatment, prevalence of resistance, and standard laboratory procedures may have changed during this time. Additionally, our analysis was limited to the identification of emerging fluoroquinolone resistance; however, these methods could be instructive for ascertainment of resistance for other important drugs, such as bedaquiline. The implementation of bedaquiline-based regimens for RR-TB treatment shows promise to advance care, but emerging resistance to this medication threatens long-term efficacy and must be identified promptly, a process that can be supported by custom linkage methods.^23^ However, these processes remain vulnerable to data entry errors, underscoring the importance of recording accurate identifiable information upon patient registration.^23–25^ Inability or failure to link patient data can negatively impact care, threaten public health by delaying diagnosis or impeding treatment initiation, and result in repeating costly laboratory testing.^19,20^

## CONCLUSION

Our study demonstrates how use of custom matching methods results in more complete and accurate linkage of patient data, a vital component to TB patient follow-up and treatment. Linkage of patient data is particularly important for longitudinal evaluation to monitor for clinical changes, such as emerging drug resistance. Having a reliable method to link these records together is important for medical providers, public health workers, and researchers to ensure that all relevant clinical information is available for them to support patient improvement.
